# CXCL12 and IL7R as Novel Therapeutic Targets for Liver Hepatocellular Carcinoma Are Correlated With Somatic Mutations and the Tumor Immunological Microenvironment

**DOI:** 10.3389/fonc.2020.574853

**Published:** 2020-12-04

**Authors:** Ke He, Shuai Liu, Yong Xia, Jianguo Xu, Fei Liu, Jing Xiao, Yong Li, Qianshan Ding, Ligong Lu, Guoan Xiang, Meixiao Zhan

**Affiliations:** ^1^ Department of General Surgery, Guangdong Second Provincial General Hospital, Guangzhou, China; ^2^ Zhuhai Interventional Medical Center, Zhuhai Precision Medical Center, Zhuhai People’s Hospital, Zhuhai Hospital Affiliated with Jinan University, Jinan University, Zhuhai, China; ^3^ Department of Biochemistry, Zhongshan School of Medicine, Sun Yat-sen University, Guangzhou, China; ^4^ Department of Hepatic Surgery, The Eastern Hepatobiliary Surgery Hospital, Navy Medical University, Shanghai, China; ^5^ Department of General Surgery, Heyuan People’s Hospital, Heyuan, China; ^6^ Department of Gastroenterology, Renmin Hospital of Wuhan University, Wuhan, China

**Keywords:** IL7R, CXCL12, somatic mutations, tumor immunological microenvironment, liver hepatocellular carcinoma

## Abstract

The mechanism of liver hepatocellular carcinoma (LIHC) development in correlation with tumor microenvironments and somatic mutations is still being elucidated. This study aims to identify the potential molecular mechanisms and candidate biomarkers in response to tumor microenvironments and somatic mutations. Multiple bioinformatics analysis methods were applied to assess the tumor immunological microenvironment, differentially expressed genes, genetic function enrichment, immunocyte infiltration, regulatory network construction, and tumor mutational burden, and to identify DNA methylation sites. The immunological microenvironment features of ESTIMATE score (OS: p = 0.017, HR = 0.64; RFS: HR = 0.43, p < 0.001) have an important impact on the prognosis of LIHC patients. Cut-off by ESTIMATE score and prognostic information identified 666 DEGs (45 downregulated and 621 upregulated) that were linked with leukocyte migration and lymphocyte activation. In immunocyte infiltration analysis, NK cells (resting), M1 macrophages, CD8+ T cells, and regulatory T cells (Tregs), which are considered core immunoregulatory cells, exhibited significant differences between higher and lower ESTIMATE scores (overall survival and recurrence-free survival p-values < 0.01). Subsequently, further analysis of immunocyte-hub gene identification illustrated that the expression levels of CXCL12 and IL7R significantly correlated with core immunoregulatory cells and somatic mutations (CXCL12: p = 2.1E-06; IL7R: p = 0.001). This study provides new insight into our understanding of the mechanisms of immunocyte regulation and microenvironment involved in LIHC development as well as the effective biomarkers of CXCL12 and IL7R and core immunoregulatory cells, which may emerge as novel therapies for LIHC patients.

## Introduction

According to estimates from the World Health Organization in 2015, cancer is one of the first two leading causes of death before age 70 in 91 of 172 countries, and it ranks third or fourth in an additional 22 countries (1). Liver hepatocellular carcinoma (LIHC) is predicted to be the sixth most commonly diagnosed cancer and the fourth leading cause of cancer death worldwide in 2018, with approximately 841,000 new cases and 782,000 deaths annually (1).

Treatment methods for liver cancer include immunotherapy, molecular targeted therapy, gene therapy, and chemotherapy (2, 3). There is currently no way to cure liver cancer in medicine (4–6). At present, the treatment of liver cancer mainly relies on surgical resection. Liver transplantation is a fundamental treatment for liver cancer. Immunotherapy for liver cancer mainly includes immunomodulators (such as interferon-α, thymosin-α1, etc.) (7), checkpoint inhibitors (such as CTLA-4 antibody, PD-1/PD-L1 antibody), and tumor vaccine (such as dendritic cell vaccine), which have been used in patients with LIHC. For patients with advanced liver cancer, chemotherapy may be considered, such as sorafenib, cisplatin, doxorubicin, and epirubicin (8–10). Therefore, it is very important to continue the study for the treatment of LIHC (9, 11).

In the present study, the occurrence of LIHC is closely related to the level of gene expression and cancer genome sequencing results; the results of a large number of preclinical *in vitro* and *in vivo* functional studies emphasize that cancer is initiated and maintained by mutations in the “driver” oncogenes and/or tumor suppressor genes that relapse. In humans, established cancers have an average of about 30–60 mutations that can change protein function, while cancers such as melanoma have about 200 mutations that change protein function per tumor (12–15). However, some cancer patients are sensitive to cancer chemotherapy drugs and have a good prognosis, which is inseparable from the changes in the tumor microenvironment (16).

Studies have shown that patients with different prognoses have different immune microenvironments and immune scores (14). The immune system can detect and eliminate abnormal or stressed cells in a variety of environments. Immune surveillance can detect cancer cells early, thereby helping to destroy early transformed cells that express new antigens. However, as cancers edit and escape the initial immunophenotypes, they also create an immunosuppressive microenvironment through direct suppression (such as cytokines, adenosine, and prostaglandins release and glucose limitation) and recruitment of cells responsible for maintaining immune tolerance to limit T cell infiltration, activation, and effector function (17). The result is an ineffective anti-tumor immune response and subsequent tumor progression. We performed a series of bioinformatics studies on the impact of the immune microenvironment on tumor prognosis in liver cancer patients with different immune microenvironments. We found that *CXCL12* and *IL7R* were novel therapeutic targets for LIHC, which correlated with somatic mutations and tumor immunological microenvironment modulation.

## Materials and Methods

### Analysis of Features of the Tumor Immunological Microenvironment

The ESTIMATE algorithm (https://bioinformatics.mdanderson.org/estimate/) was applied to calculate the tumor purity and immune infiltration level using gene expression signatures (18). This algorithm can detect the stromal and immune score of each sample to predict the tumor stromal and immune infiltration and thus construct the ESTIMATE score, a microenvironment comprehensive score, to reflect tumor purity in cancer samples (18). The RNA sequencing (RNA-seq) fragments per kilobase of exon per million fragments mapped reads (FPKM) of the TCGA-LIHC project were downloaded from the “TCGAbiolinks” R package (19). Here, each sample’s stromal, immune score, and estimate score of TCGA-LIHC were calculated by the ESTIMATE algorithm using the “ESTIMATE” R package. The “survminer” package was applied to perform Kaplan–Meier survival analysis of the stromal, immune, and ESTIMATE scores with a continuous scale and to determine an optimal cut-off point for each variable using the smoothHR (Smooth Hazard Ratio Curves Taking a Reference Value) algorithm. This is to quantify the risk ratio of continuous variables at each value, by referring the baseline (cut-off point) as the criterion based on multiple Cox proportional hazards regression used in restricted cubic splines (20).

### Data Processing and Identification of Co-Differentially Expressed Genes

Based on the cut-off point of the ESTIMATE score, we divided the TCGA-LIHC tumor samples into high and low risk groups. Subsequently, to facilitate comparative analysis, we transformed the FPKM data into transcripts per kilobase million (TPM) (21, 22) with the following formula: $\mathrm{TP}M_{i}=\left(\frac{\mathrm{FPKMi}}{\sum_{\text{j}}\mathrm{FPKMj}}\right)\times 10^{6}$. Differential expression analysis was performed by the “DESeq2” R package for TPM normalized TCGA-LIHC data (5). For multiple probes mapped to the same gene symbol, the average value was considered as the gene expression value (23–25). In light of statistical effectiveness, adjusted p-value of the false discovery rate (FDR) was corrected with Benjamini–Hochberg method, and the gene expression fold change (FC) was subsequently detected. In the present study, genes that satisfied the standard criteria of |log2FC| > 1.5 and adjusted p-value < 0.05 were considered differentially expressed genes (DEGs) in the TCGA-LIHC project (24, 25). Following extraction, the clustering heatmap was drawn for enriched genes of the hub pathway in samples from the TCGA-LIHC project and thus determined as hub DEGs.

### Genetic Function Enrichment Analysis

We used the “clusterprofiler” R package to annotate and visualize the Gene Ontology (GO) functional enrichment, which extracts comprehensive biological knowledge based on large lists of genomic studies and databases to categorize the DEGs into biological process (BP), molecular function (MF), and cellular composition (CC) categories (26, 27). The GO terms associated with p < 0.05 were considered to be significantly enriched.

Following selection of DEGs, we performed an extensive functional enrichment analysis using the online tool of the Metascape database (http://metascape.org/gp/index.html#/main/step1) to construct a Kyoto Encyclopedia of Genes and Genomes (KEGG) pathway network, with a cut-off criteria of p < 0.05 and enrichment score > 3.0 (28). In addition, Cytoscape software (V3.5.1; http://cytoscape.org/) was used to visualize and evaluate interactions of the KEGG pathway network (29). After extracting the higher degree regulators, we constructed an automatic Kaplan–Meier survival analysis, based on the “maxstat” algorithm, to visualize and evaluate prognostic effects and identify hub genes for subsequent analysis.

### Immunocyte Infiltration and Regulatory Network Analysis

To characterize prognostic immune cell subsets of TCGA-LIHC, we applied CIBERSORT estimate software to quantify the immune cell fractions for the gene expression matrix derived from TCGA-LIHC cancer samples. The advantage of the CIBERSORT algorithm is combining the ν-support vector regression (ν-SVR) method that maximally reduced the data noise from high-dimensional genomic matrix, and thus accurately captures the infiltration of various immune cell subtypes (30). We also performed a Kaplan–Meier survival analysis for immune cell subtypes to detect prognostic corrected cell subtypes. Subsequently, the hub immunocytes with the significant expression differences and survival significance were extracted based on immunocyte infiltration analysis of TCGA-LIHC tissue expression profiles and the immunocytes detection. In addition, Spearman correlation analysis was used to calculate cell subtype correlations and p-values. Based on these correlation values, we used a graph algorithm (igraph: https://github.com/igraph) to construct the immune cell association network.

### Detection of Tumor Mutational Burden and DNA Methylation Sites

To gain further insight into the epigenetic modification sites of DNA methylation, we applied an intuitive web tool of Wanderer (http://maplab.imppc.org/wanderer/) to identify potential sites at which the methylation level was negatively correlated with transcriptional expression (31). Here, the data type of 450K methylation array was selected, and the sites with adjusted p-value considered as different loci.

Additionally, the tumor mutational burden (TMB) involved in LIHC pathogenesis in response to expression of the hub genes, the somatic called variants were analyzed by “mutect2” pipeline that determined TCGA raw mutation counts, and 35Mb size exome was used as the reference genome. Additionally, we further performed a somatic mutation analysis based on the Wilcoxon signed-rank test. The TCGA-LIHC tumor sample somatic variants were obtained from “TCGAbiolinks” as the raw mutation count. For the estimate, the files were aligned to the genome of hg38 GRCh38 (32).

### Prediction of Transcription Factors of Candidate Genes

To predict the potential transcription factors regulating the candidate genes, we reanalyzed the ATAC-seq data of the TCGA-LIHC project using UCSCXenaTools (https://cran.rproject.org/web/packages/UCSCXenaTools/vignettes/USCSXenaTools.html). UCSCXenaTools is an R package for genomics data combination and exploration, and it was download on an integrated platform of public databases such as TCGA, ICGC, TARGET, GTEx, CCLE, et al. (33). After calling the genomic peak and DEGs, we present the top five highest coefficients of correlation regulators in correlation with candidate genes as potential regulators.

### Validation of Hub Gene Expression and Clinicopathological Variables

To validate the results, five cancer and five paracancerous tissues were obtained from five LIHC patients undergoing liver cancer surgery at the Guangdong Second Provincial General Hospital. These patients were diagnosed with LIHC. This research was conducted under the approval and supervision of the Ethics Committee of Guangdong Second Provincial General Hospital. Total RNA was extracted from the tissues using Trizol (Invitrogen, Carlsbad, CA, USA) according to the manual instructions. Samples of 60 mg tissue were grinded with liquid nitrogen into powder and transferred into a 2 ml tube containing 1.5 ml Trizol reagent. The mixture was centrifuged at 12000×g for 5 min at 4°C. The supernatant was transferred to a new 2 ml tube, and 0.3 ml of Chloroform/isoamyl alcohol (24:1) per 1.5 ml of Trizol reagent was added. After the mixture was centrifuged at 12000×g for 10 min at 4°C, the aqueous phase was transferred to a new 1.5 mL tube that was added with equal volume of isopropyl alcohol. The mix was centrifuged at 12000×g for 20 min at 4°C and the supernatant was removed. After being washed with 1 ml 75% ethanol, the RNA pellet was air-dried in the biosafety cabinet and then dissolved by adding 25-100 μl DEPC-treated water. Subsequently, total RNA was qualified and quantified using a Nano Drop and Agilent 2100 bioanalyzer (Thermo Fisher Scientific, MA, USA).

According to the manufacturer’s instructions, the first step involves the removal of ribosomal RNA (rRNA) using target-specific oligos and RNase H reagents to eliminate both cytoplasmic (5S rRNA, 5.8S rRNA, 18S rRNA, and 28S rRNA) and mitochodrial ribosomal RNA (12S rRNA and 16S rRNA) from total RNA preparations. Following SPRI beads purification, the RNA is fragmented into small pieces using divalent cations under elevated temperature. The cleaved RNA fragments are copied into first strand cDNA using reverse transcriptase and random primers, followed by second strand cDNA synthesis using DNA Polymerase I and RNase H. This process removes the RNA template and synthesizes a replacement strand, incorporating dUTP in place of dTTP to generate ds cDNA. These cDNA fragments then have the addition of a single ‘A’ base and subsequent ligation of the adapter. After UDG treatment, the incorporation of dUTP quenches the second strand synthesis during amplification. The products were enriched by PCR to create the final cDNA library. The libraries were assessed on quality and quantity in two methods: Agilent 2100 bioanalyzer was used to check the distribution of the fragments size, and real-time quantitative PCR (QPCR) (TaqMan Probe) was used to quantify the library. Pair-end sequence of the qualified libraries was performed on the Illumina Hiseqvoseq by Jiayin Biotechnology Ltd (Shanghai, China).

### RNA-Seq Analysis

First, effective reads sequence was extracted while index sequence and adapter sequence in each sequence were removed. Thereafter, RNA-seq data was mapped to hg19 using hisat2 and cufflinks to calculate the gene expression level in all samples. We analyze mRNA coverage and characteristics, including box-and-whisker diagram analysis of the coverage of reads in different regions and the distribution of reads on homogenized mRNA. At last, Quantile normalizations of the gene expression level were performed. FDR < 0.1 and |log2 (fold change) > 1 were set as the cutoff for DEGs.

### Immunohistochemical Staining

All 42 LIHC samples and paired non-neoplastic tissues used in immunohistochemical were retrieved from the Department of Pathology, Guangdong Second Provincial General Hospital, China. Before they were used, all cases were diagnosed by two certificated pathologists without discrepancy. This research was conducted under the approval and supervision of the Ethics Committee of Guangdong Second Provincial General Hospital. The paraffin-embedded tissues were first stained with hematoxylin and eosin (HE) for histological examination. Subsequently, deparaffinized sections were treated with 3% H_2_O_2_ and subjected to antigen retrieval by citric acid (pH 6.0). After overnight incubation with primary antibody (anti-CXCL12 and IL7R antibody; Boster Bio, Pleasanton, USA) by 1:200 at 4°C, sections were incubated for 15 min at room temperature with horseradish peroxidase-labeled polymer conjugated with secondary antibody (MaxVision Kits) and incubated for 1 min with diaminobenzidine. The sections were then lightly counterstained with hematoxylin. The sections without primary antibody were served as negative controls. Expression level of CXCL12 and IL7R was determined according to the average score of two pathologists’ evaluations.

### RNA Isolation and Real-Time PCR Analysis

ALL 80 LIHC tissue samples and paired non-neoplastic tissues (stored at -80°C) used for RT-PCR detection were randomly selected from the clinical sample library of the Guangdong Second Provincial General Hospital. Total RNA was isolated from cell lines or tissues with TRIzol reagents (Invitrogen) according to the manufacturer’s instructions. RT-PCR was performed to mRNA expression with SYBR Green PCR Master Mix (TaKaRa, Ohtsu, Japan). β-actin was used for CXCL12 and IL7R normalization. Expression of transcripts was assessed using the following primers: CXCL12: Forward 5’-TGAGAGCTCGCTTTGAGTGA-3’, Reverse 5’- CACCAGGACCTTCTGTGGAT-3’; IL7R: Forward 5’- ATCGCAGCACTCACTGACCTGT-3’, Reverse 5’- TCAGGCACTTTACCTCCACGAG -3’.

## Results

### LIHC Immunological Microenvironment Features

**Figure 1:**
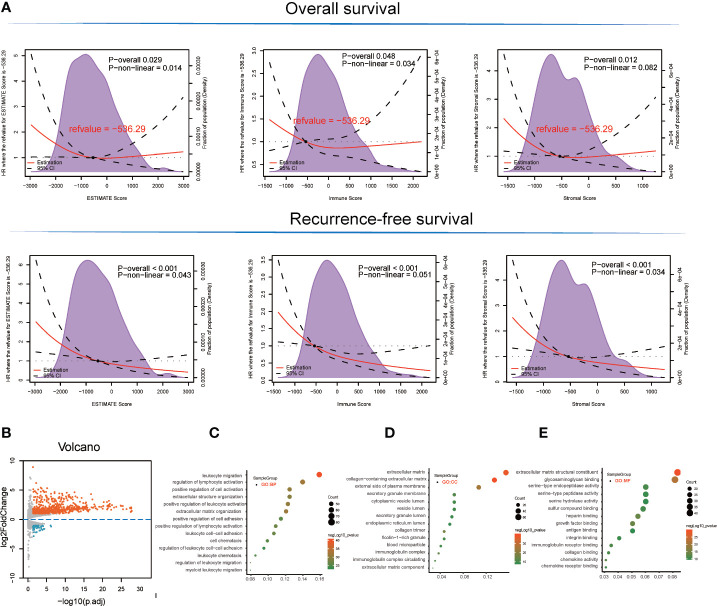
Survival analysis and detection of immunological microenvironment-related differentially expressed genes (DEGs) as immunological microenvironment features. **(A)** Overall survival and recurrence-free survival analyses of stromal, immune, and ESTIMATE scores based on TCGA-LIHC tissues. **(B)** Volcano plot showing immunological microenvironment-related DEGs of the ESTIMATE score in TCGA-LIHC tissues. **(C–E)** GO analysis, including BP, MF, and CC enrichment with respect to immunological microenvironment-related DEGs of the ESTIMATE score.

With respect to the TCGA-LIHC microenvironment score, we performed Kaplan–Meier survival analysis using the TCGA-LIHC transcriptional expression profile. Our results showed that the immunological microenvironment features, including the stromal score (overall survival [OS]: hazard ratio [HR] = 0.52, p < 0.001; recurrence-free survival [RFS]: HR = 0.52, p < 0.001), immune score (OS: p = 0.175, HR = 0.78; RFS: HR = 0.45, p < 0.001), and ESTIMATE score (OS: p = 0.017, HR = 0.64; RFS: HR = 0.43, p < 0.001) positively correlated with a better prognosis in LIHC patients (**Figure 1A** and **Table S1**).

### Microenvironment Score-Related Differential Gene Detection

Based on ESTIMATE score and corresponding patient’s prognostic information, we detected the cut-off value of −536.29 and thus grouped LIHC sample into high and low score groups. Consequently, 666 DEGs (45 downregulated and 621 upregulated) were identified in the comparison between high and low ESTIMATE score samples in TCGA-LIHC patients (**Figure 1B** and **Table S2**) following the LIMMA powers differential expression analyses.

### DEG Functional Enrichment Analysis

GO term enrichment analysis of the DEGs identified from TCGA-LIHC produced the following results (1). With respect to ESTIMATE score-related DEG enriched GO terms, the BP terms GO:0050900~leukocyte migration (gene count = 82, p = 3.42E-41), GO:0051249~regulation of lymphocyte activation (gene count = 72, p = 1.08E-32), and GO:0030198~extracellular matrix organization (gene count = 62, p = 4.08E-32) were significantly enriched. GO:0062023~collagen-containing extracellular matrix (gene count = 69, p = 1.06E-40), GO:0031012~extracellular matrix (gene count = 79, p = 3.74E-38), and GO:0009897~external side of plasma membrane (gene count = 55, p = 1.57E-25) terms were identified in the CC category while the MF terms GO:0005201~extracellular matrix structural constituent (gene count = 43, p = 1.91E-30), GO:0005539~glycosaminoglycan binding (gene count = 42, p = 1.19E-22), and GO:0019838~growth factor binding (gene count = 26, p = 1.63E-22) were significantly enriched (**Figures 1C–E** and **Table S3**).

### Immunocyte Infiltration Detection and Survival Analysis

**Figure 2:**
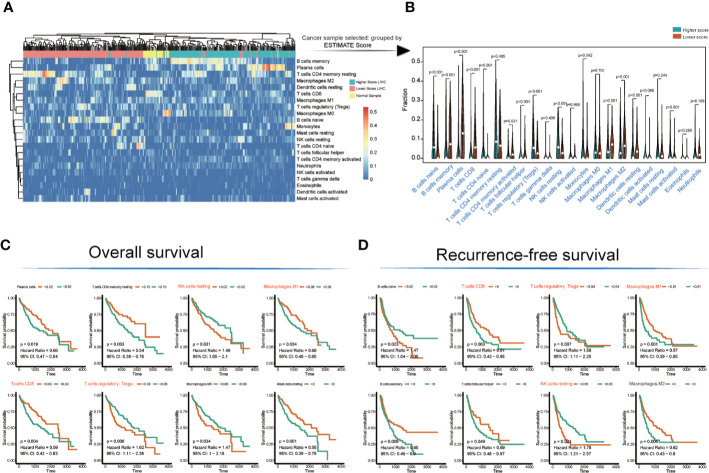
Immunocyte infiltration and survival analyses of cancer tissue immunocytes. **(A, B)** Immunocyte infiltration and differential analysis of TCGA-LIHC tumor samples and normal tissues. **(C, D)** Overall survival and recurrence-free survival analyses of infiltrated immunocytes.

The landscape of immunocyte infiltration in response to LIHC cancer and paracancerous sample were detected with CIBERSORT algorithm (**Figure 2A**). Further analysis based on statistical analysis between the high and low ESTIMATE score LIHC samples identified a series of important immunocyte subtypes in the tumor microenvironment in immunocyte infiltration analysis (**Figure 2B**), including naïve B cells (p < 0.001), memory B cells (p < 0.001), plasma cells (p < 0.001), CD8+ T cells (p < 0.001), CD4 naive T cells (p < 0.001), CD4 memory activated T cells (p = 0.031), follicular helper T cells (p < 0.001), regulatory T cells (Tregs) (p < 0.001), NK cells (resting) (p < 0.001), monocytes (p = 0.042), M1 macrophages (p < 0.001), M2 macrophages (p < 0.001), resting dendritic cells (p < 0.001), and activated mast cells (p < 0.001). The LIHC sample information and corresponding immune cell infiltration value were presented in **Table S4**.

However, not all immunocyte subtypes had an effect on the prognosis of LIHC patients. Furthermore, OS and RFS analyses revealed that the following cell population exhibited significant differences between high and low ESTIMATE-scored groups (**Figures 2C, D**): NK cells (resting) (OS: p = 0.031, HR = 1.49; RFS: p < 0.001, HR = 1.76), M1 macrophages (OS: p = 0.034, HR = 0.66; RFS: p = 0.001, HR = 0.57), CD8^+^ T cells (OS: p = 0.004, HR = 0.59; RFS: p = 0.003, HR = 0.61), and Tregs (OS: p = 0.006, HR = 1.62; RFS: p = 0.007, HR = 1.58).

### Construction of the Immune Cell Regulatory Network

**Figure 3:**
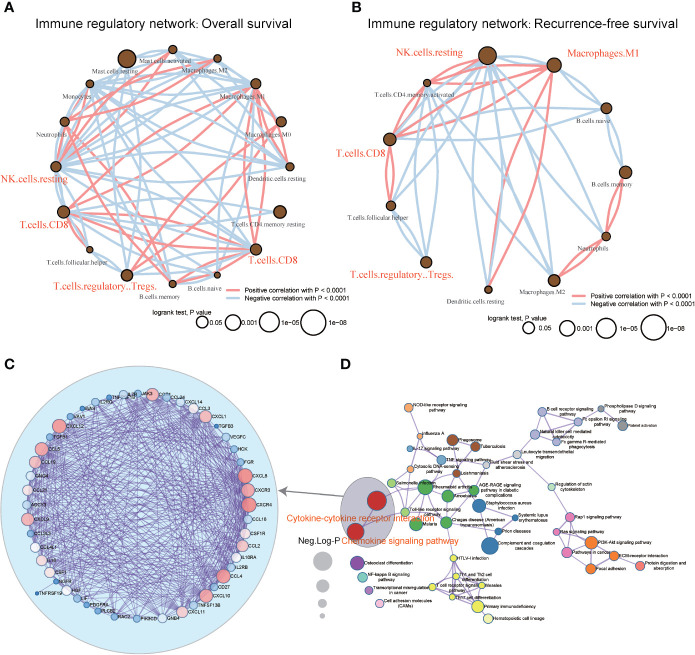
Construction of immunocyte regulatory and KEGG. pathway networks with respect to the LIHC immunological microenvironment **(A, B)** Immunocyte infiltration regulatory network analysis of TCGA-LIHC data in response to overall and recurrence-free survival. The dot size represents the log rank test p-value. **(C)** After filtering the genes in primarily enriched pathway terms, the hub gene PPI network was generated. The size of the font represents the node degree, and the color of the dots represents the closeness-centrality. **(D)** The network of KEGG pathways was identified using the MetaScape database. The sizes of the dots represent the enrichment score.

The TCGA-LIHC immune cell infiltration value was calculated to construct the immune cell regulatory network based on supervised clustering analysis in response to the OS and RFS analyses (**Figures 3A, B** and **Table S5**). At present, the infiltration subtypes of NK cells (resting) (OS: HR = 1.49, 95% confidence interval [CI] = 1.05–2.1, p = 0.031; RFS: HR = 1.76, 95% CI = 1.21–2.57, p = 0.0001), M1 macrophages (OS: HR = 0.66, 95% CI = 0.46–0.95, p = 0.034; RFS: HR = 0.57, 95% CI = 0.39–0.85, p = 0.001), CD8^+^ T cells (OS: HR = 0.66, 95% CI = 0.47–0.94, p = 0.019; RFS: HR = 0.61, 95% CI = 0.43–0.85, p = 0.003), and Tregs (OS: HR = 1.62, 95% CI = 1.11–2.38, p = 0.006; RFS: HR = 1.58, 95% CI = 1.11–2.225, p = 0.007) were selected as key regulators (**Figures 3A, B** and **Table S5**).

### Hub Genes Identified in Response to Immunological Microenvironment Features

Here, the 666 DEGs were selected to construct a KEGG pathway network, and hsa04060~Cytokine-cytokine receptor interaction (gene count = 39, Neg.Log p = 19), hsa04062~Chemokine signaling pathway (gene count = 30, Neg.Log p = 16), and hsa04610~Complement and coagulation cascades (gene count = 21, Neg.Log p = 16) were primarily detected (**Figure 3D** and **Table S6**).

**Figure 4:**
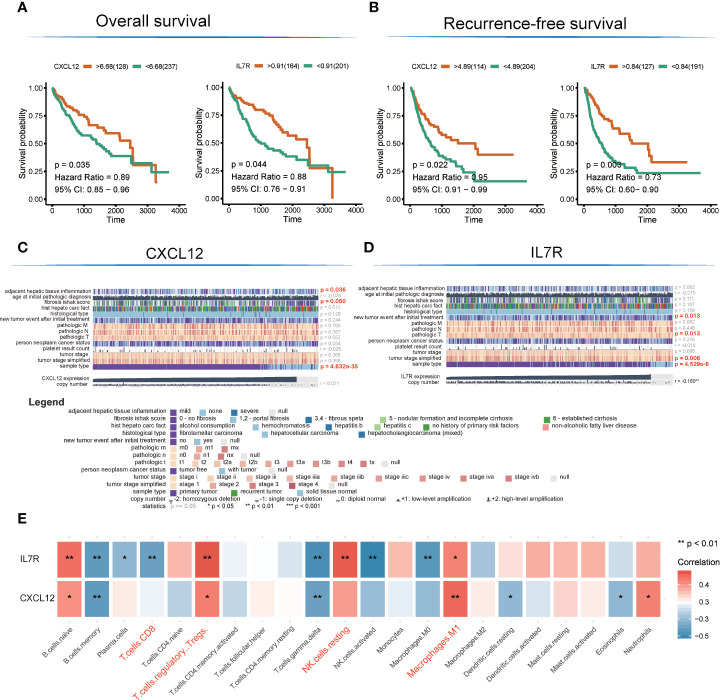
Detection of hub genes and correlation analysis among hub genes, clinical characteristics, and immunocyte infiltration. **(A, B)** Kaplan–Meier overall survival and recurrence-free survival analyses of CXCL12 and IL7R gene expression. **(C, D)** Heatmap showing correlation analysis of LIHC clinical characteristics based on clinical information of TCGA-LIHC patients **(E)**. Correlation analysis among hub genes and infiltrated immunocytes.

There are altogether 51 genes enriched in hsa04060~Cytokine-cytokine receptor interaction and hsa04062~Chemokine signaling pathway, which were extracted to construct the protein-protein interaction (PPI) network and to perform OS and RFS survival analyses. The PPI network was presented in **Figure 3C**. Additionally, 7 genes shown a significant influence on OS survival, and 4 genes presented a significant influence on RFS survival for LIHC patients (**Figure S1**). *CXCL12* (degree = 21; OS: p = 0.035, HR = 0.89; RFS: p = 0.022, HR = 0.91) and *IL7R* (degree = 19; OS: p= 0.003, HR = 0.73; RFS: p =0.044, HR = 0.88) enjoyed relative higher connective degree and statistical significance than other nodes in response to OS and RFS, and were selected as candidate biomarkers (**Figures 4A, B**).

The expression level of *CXCL12* was also correlated with tumor adjacent hepatic tissue inflammation (p = 0.036), fibrosis Ishak score (p = 0.05), and tumor sample type (p = 4.632E-35), while the expression of *IL7R* was involved in new tumor events after initial treatment (p = 0.013), tumor pathologic stage (T stage; p = 0.013), tumor stage simplified (p = 0.006), and tumor sample type (p = 4.53E-6) (**Figures 4C, D**).

**Figure 5:**
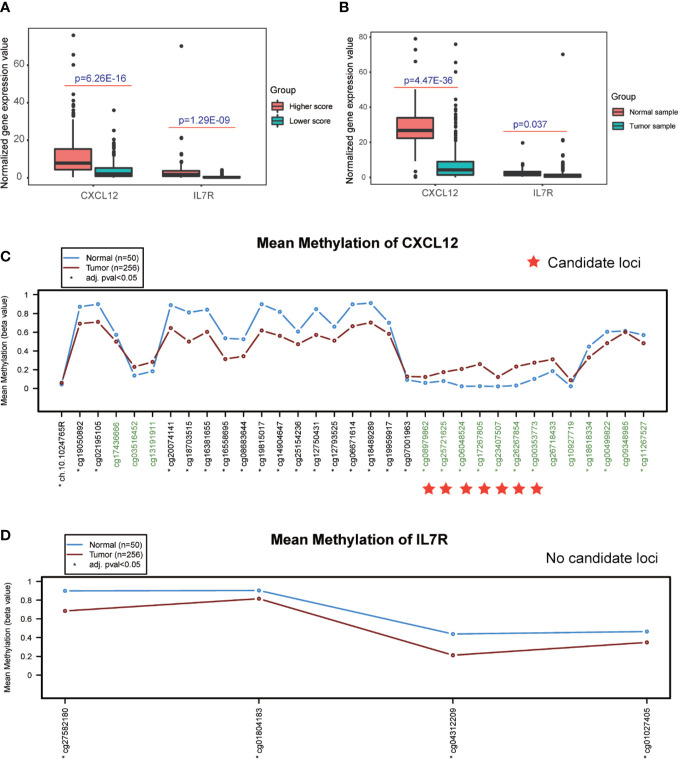
Detection of differential expression and DNA methylation sites. **(A, B)** Differential expression analysis of higher and lower ESTIMATE scores, and different tumor samples. **(C, D)** DNA methylation sites in response to the CXCL12 and IL7R gene region.

Interestingly, the expression levels of *CXCL12* and *IL7R* also significantly correlated with the infiltration value of NK cells (resting) (*CXCL12*: p = 0.062; *IL7R*: p = 7.37E-05), M1 macrophages (*CXCL12*: p = 0.0025; *IL7R*: p = 0.018), CD8^+^ T cells (*CXCL12*: p = 0.798; *IL7R*: p = 0.0035), and Tregs (*CXCL12*: p = 0.014; *IL7R*: p = 0.0008) (**Figure 4E** and **Table S7**). *CXCL12* and *IL7R* also exhibited significant differences in expression level between normal and tumor tissues (*CXCL12*: p = 4.47E-36; *IL7R*: p = 0.037), and between high and low ESTIMATE-scored groups (*CXCL12*: p = 6.26E-16; *IL7R*: p = 1.29E-09) (**Figures 5A, B**).

### Detection of TMB, DNA Methylation Sites, and Transcription Factors of Candidate Genes

The DNA methylation site results illustrated that *CXCL12*-related sites, which include cg08979862, cg25721625, cg06048524, cg17267805, cg23407507, cg26267854, and cg00353773, were differentially methylated in the comparison between normal and tumor tissues, while no sites were detected in the *IL7R* gene region (**Figures 5C, D** and **Table S9**).

**Figure 6:**
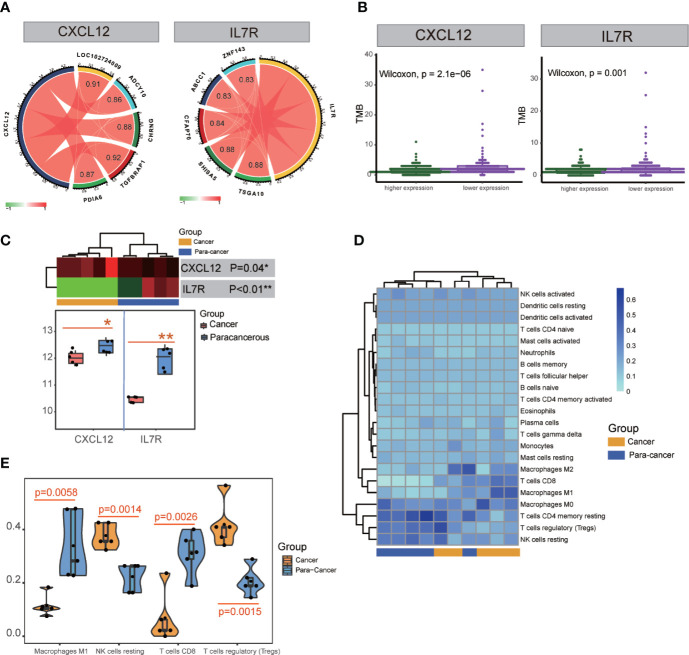
TMB and transcription factor detection, and hub gene expression and immunocyte infiltration validation. **(A)** Identification of transcription factors correlated with CXCL12 and IL7R gene expression. TSGA10, SHISA5, CFAP70, ABCC1, and ZNF143 were detected for IL7R; TGFBRAP1, LOC102724009, CHRNG, PDIA6, and ADCY10 were detected for CXCL12. **(B)** TMB analysis of CXCL12 and IL7R expression. **(C)** Significant differences in the expression of CXCL12 and IL7R in cancerous and paracancerous tissues were detected in RNA-seq analysis. **(D, E)** Significant differences among cancerous and paracancerous samples for NK cells (resting), M1 macrophages, CD8+ T cells, and Tregs in the immunocyte infiltration validation.

TMB is a clinically validated biomarker involved in antitumor immunity that functions to drive T-cell responses. As shown in **Figure 6B** and **Table S8**, there was a significant difference between better prognosis and expression levels of *CXCL12* and *IL7R* (*CXCL12*: p = 2.1E-06; *IL7R*: p = 0.001). Upregulated CXCL12 and IL7R expression is associated with better prognosis. And both CXCL12 and IL7R expression were negatively associated with TMB, indicating a negative relationship.

ATAC-seq analysis revealed the transcription factors *TSGA10* (correlation index = 0.88), *SHISA5* (correlation index = 0.88), *CFAP70* (correlation index = 0.84), *ABCC1* (correlation index = 0.83), and *ZNF143* (correlation index = 0.83) were significantly correlated with *IL7R* expression, while *TGFBRAP1* (correlation index = 0.92), *LOC102724009* (correlation index = 0.91), *CHRNG* (correlation index = 0.88), *PDIA6* (correlation index = 0.87), and *ADCY10* (correlation index = 0.86) were significantly correlated with *CXCL12* expression (**Figure 6A** and **Table S10**).

### Validation of Hub Gene Expression and Immunocyte Infiltration

Five patients’ clinicopathologic characteristics are listed in **Table S11**. As shown in **Figure 6C**, the RNA-seq data showed a significant difference in *CXCL12* expression between cancerous and paracancerous tissues (p = 0.04), as well as the *IL7R* expression (p<0.01). There was also a significant difference between cancerous and paracancerous samples in NK cells (resting) (p = 0.0014), M1 macrophages (p = 0.0058), CD8^+^ T cells (p = 0.0026), and Tregs (p = 0.0015) in the immunocyte infiltration analysis (**Figures 6D, E**).

### *CXCL12* and *IL7R* Are Downregulated in LIHC on Both mRNA and Protein Levels

**Figure 7:**
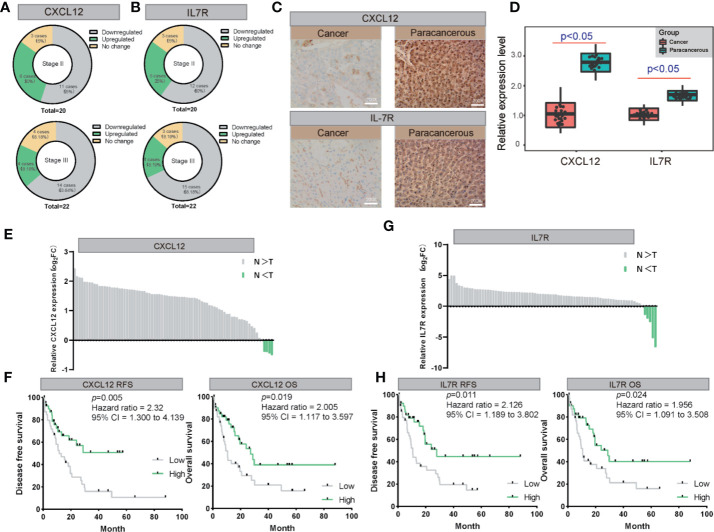
The validation of both of CXCL12 and IL7R expression in LIHC on both mRNA and protein levels. **(A, B)** The expression of CXCL12 and IL7R was downregulated in most LIHC patients examined by immunohistochemical staining. **(C, D)** Representative photographs of staining of CXCL12 and IL7R protein in a pair of LIHC and adjacent tissues (200×). **(E, F)** Lysates from paired tissues of LIHC and adjacent tissue were analyzed by real-time PCR for the detection of CXCL12. Low expression of CXCL12 is associated with poor prognosis of LIHC. Low CXCL12 mRNA levels reduce OS and RFS of LIHC patients. **(G, H)** Lysates from paired tissues of LIHC and adjacent tissue were analyzed by real-time PCR for the detection of IL7R. Low expression of IL7R is associated with poor prognosis of LIHC. Low IL7R mRNA levels reduce OS and RFS of LIHC patients.

We also examined the expression levels of *CXCL12* and *IL7R* in 42 LIHC patients and corresponding noncancerous liver tissues using immunohistochemical staining. *CXCL12* was expressed in cytoplasm and *IL7R* was expressed in membrane (**Figures 7A, B**), which was consistent with previous reports. Our results indicated that *CXCL12* and *IL7R* are down-regulated in most LIHC patients, and the down-regulation of *CXCL12* and *IL7R* in stage-II LIHC patients is 55% and 60%, and the down-regulation of *CXCL12* and *IL7R* in stage-III LIHC patients is 63.64% and 68.18% (**Figures 7C, D**).

*CXCL12* and *IL7R* expressions of 80 randomly selected LIHC tissues paired with paracancerous liver tissues were investigated by quantitative real-time PCR (qRT-PCR). From this, all 80 LIHC tissues exhibited significant down-regulation of *CXCL12* and *IL7R* in LIHC (**Figures 7E, G**). These findings evidently demonstrated that *CXCL12* and *IL7R* was downregulated in LIHC on both mRNA and protein level, implying the importance of it in LIHC pathogenesis.

Importantly, Kaplan-Meier survival analysis showed 80 LIHC patients with tumors displaying low *CXCL12* expression levels had significantly shorter overall survival (OS) (P = 0.019, hazard ratio = 2.005, 95% CI = 1.117–3.597) and recurrence-free survival (RFS) (P = 0.005, hazard ratio = 2.31, 95% CI = 1.300–4.139) compared to those with high *CXCL12* expression tumors **Figure 7F**). In addition, Kaplan-Meier survival analysis showed among the 80 LIHC patients, lower *IL7R* expression levels correlated with significantly shorter overall survival (OS) (P = 0.024, hazard ratio = 1.956, 95% CI = 1.091–3.508) and recurrence-free survival (RFS) (P = 0.011, hazard ratio = 2.126, 95% CI = 1.189–3.802) compared to those with high *IL7R* expression (**Figure 7H**).

## Discussion

Studies have shown that the tumor microenvironment plays a vital role in checkpoint inhibitor immunotherapy (34, 35), and immune cells are closely related to tumor development (36–38). This study found that tumor patients with different immune scores have different survival rates, different immune subcellular composition, and a correlation between immune cells and immune-related genes (**Figures 2A, B**) (39). The overall survival rate and recurrence-free survival rate of patients with high immune scores were better than those of the low score group (**Figures 4A, B**). According to the single immune subcellular score, it was found that the immune subcellular score would affect the overall survival rate and recurrence-free survival rate (**Figures 2C, D**). Using KEGG signaling pathway analysis, two pathways were found to regulate immune function (**Figures 3C, D**), and the downstream genes *CXCL12* and *ILR7* were studied. We found that the immune score is related to the expression levels of *CXCL12* and *ILR7*. The chemokine *CXCL12*/stromal cell-derived factor 1 is essential for the migration of leukocytes to lymphoid organs and inflamed tissues, and stimulates tumor development (40).

We further studied the relationship between the expression of *CXCL12* and *IL7R* and the overall survival rate and recurrence-free survival rate. The results showed that the OS and RFS of the high score group were higher than the low group (**Figures 4A**, **B**). Subsequently, we divided the expression levels of *CXCL12* and *IL7R* into a high expression group and a low expression group. It was suggested that the expression levels of these genes were related to the distribution of core immune cells (**Figures 4C, D**). Analysis of the immune scores and expression levels of *CXCL12* and *IL7R* also showed that in the group with higher immune scores, the expression levels of *CXCL12* and *IL7R* were higher (**Figure 5A**), and immune cells were strongly correlated with *CXCL12* and *IL7R* (**Figure 4E**), indicating that the immune microenvironment affects gene expression levels. The expression of *CXCL12* and *IL7R* in normal patients is higher than that of LIHC patients (**Figure 5B**), indicating that *CXCL12* and *IL7R* are associated with tumorigenesis, but whether high expression can inhibit tumorigenesis and development, while low expression can promote tumor progression, is to be further discussed. This result has also been verified at the level of gene methylation (**Figure 5C**). It is generally believed that higher methylation level of a gene corresponds to lower gene expression level (36, 41), so observing the methylation level of a gene may indirectly assess the expression level. We found that the methylation level of *CXCL12* gene in normal tissues was lower than that in tumor tissues, indicating that the expression level of *CXCL12* in normal tissues was higher than that in tumor. In addition, by studying the relationship between the expression of *CXCL12*, *IL7R*, and TMB, we found higher TMB levels when lower *CXCL12* and *IL7R* gene expression levels were observed (**Figure 6B**), indicating that high *CXCL12* and *IL7R* expression inhibited TMB occurrence, but the specific mechanism is not yet clear.

In order to further explore the study, we collected cancer and paracancerous samples to analyze the composition of immune cells and the expression of *CXCL12* and *IL7R*. The result is that the difference between the immune microenvironment and the composition of immune cells in cancer and paracancerous tissues was discussed earlier. The results are roughly consistent, confirming the difference in the expression of *CXCL12* and *IL7R* genes between cancerous and paracancerous tissues (**Figures 6C, D**).

These results suggested that immune microenvironments affect the occurrence of tumors by affecting the immune cell compositions. However, further research is needed to explore the pathways through which genes affect the tumor immune microenvironment. *CXCL12* and *IL7R* may be used as a new therapeutic target to study how different immune microenvironments affect cancer pathogenesis and how these genes regulate the interaction between different immune cells and the tumor microenvironment.

In summary, we performed a systematic bioinformatics analysis of potential molecular mechanisms and candidate biomarkers that correlated with the LIHC tumor microenvironment and somatic mutations. We found that NK cells (resting), M1 macrophages, CD8+ T cells, and Tregs were involved in generation of the LIHC tumor microenvironment. Additionally, the maps of leukocyte migration and lymphocyte activation correlated with LIHC tumor microenvironment construction. The expression levels of *CXCL12* and *IL7R* significantly correlated with the construction of the tumor microenvironment and somatic mutations, and they are identified as potential diagnostic and therapeutic targets in the LIHC.

## Data Availability Statement

The datasets presented in this study can be found in online repositories. The names of the repository/repositories and accession number(s) can be found below: NCBI Gene Expression Omnibus [accession: GSE154964].

## Ethics Statement

The studies involving human participants were reviewed and approved by the Ethics Committee of Guangdong Second Provincial General Hospital. The patients/participants provided their written informed consent to participate in this study. Written informed consent was obtained from the individual(s) for the publication of any potentially identifiable images or data included in this article.

## Author Contributions

KH, SL, YX, and JX performed the experiments and analyzed the data. FL, QD, and JX contributed reagents and materials. KH, MZ, and GX conceived and designed the experiments. HK, YL, LL, JX, and GX discussed the manuscript. GX critically reviewed the manuscript. KH, MZ, and GX wrote the paper. All authors contributed to the article and approved the submitted version.

## Funding

This work was financed by the National Key Research and Development Program of China (No. 2017YFA0205200), National Natural Science Foundation of China (No. 81901857 and 81771957), Natural Science Foundation of Guangdong Province, China (No. 2018A030313074), Youth Science Foundation of Guangdong Second Provincial General Hospital (No. YQ2016-001), Foundation of Shanghai Municipal Health Commission (No. 2018BR34), and 3D Printing Research Project (2020) of Guangdong Second Provincial General Hospital (No. 3D-A2020001).

## Conflict of Interest

The authors declare that the research was conducted in the absence of any commercial or financial relationships that could be construed as a potential conflict of interest.
