# Supplementary figures and images for: CXCL12 and IL7R as Novel Therapeutic Targets for Liver Hepatocellular Carcinoma Are Correlated With Somatic Mutations and the Tumor Immunological Microenvironment
Source: Front Oncol. 2020 Dec 4;10:574853. doi: 10.3389/fonc.2020.574853 (PMC7746863; doi:10.3389/fonc.2020.574853)

# LHC RFS analysis

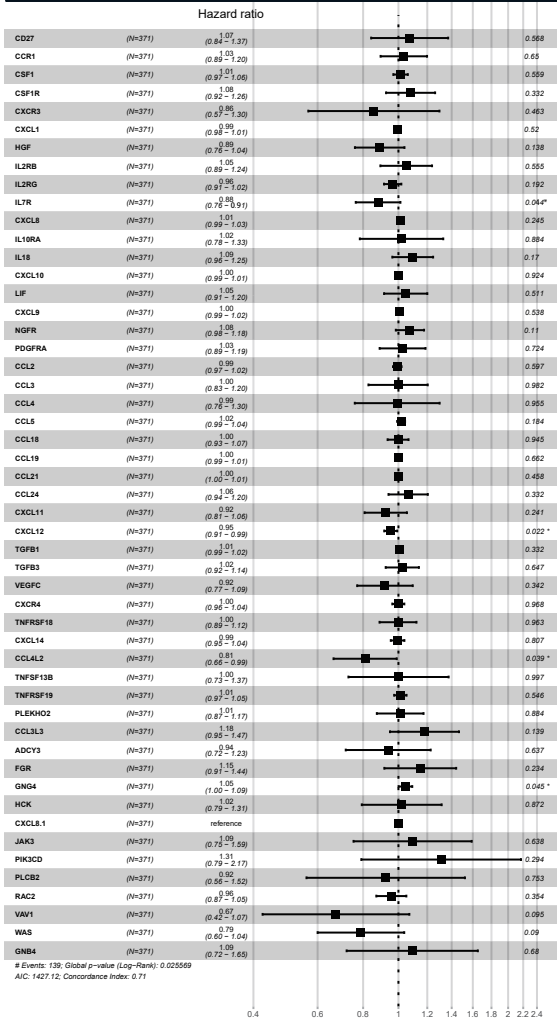

# LHC OS analysis

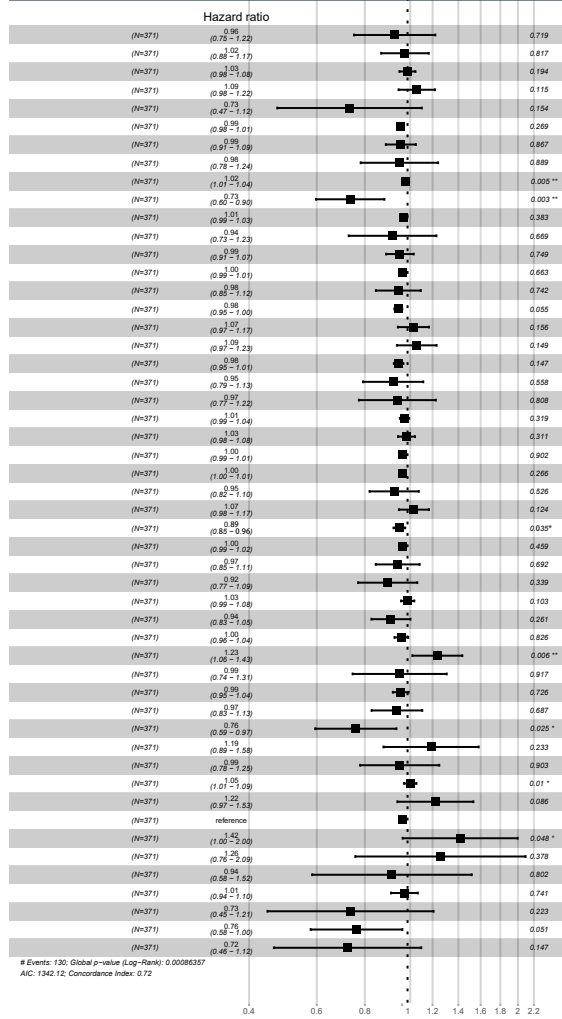

Supplement: Supplementary file 1 [file DataSheet_1.zip › Additional file/Figure S1_Hub gene OS and RFS analysis.pdf]
